# Uncovering the essence of moving experiences in Japanese culture: Development and validation of a *kando* reaction scale

**DOI:** 10.1371/journal.pone.0311905

**Published:** 2024-12-05

**Authors:** Haruka Shoda, Shoko Yasuda, Ai Uemiya, Atsushi Yuhaku, Tadao Isaka

**Affiliations:** 1 Department of Musicology, Faculty of Music, Kyoto City University of Arts, Kyoto, Japan; 2 Mori Arinori Institute for Higher Education and Global Mobility, Hitotsubashi University, Kunitachi, Japan; 3 Institute of Human and Social Sciences, Kanazawa University, Kanazawa, Japan; 4 Faculty of Sport and Health Science, Ritsumeikan University, Kusatsu, Japan; 5 Institute of Advanced Research for Sport and Health Science, Ritsumeikan University, Kusatsu, Japan; Southeast University, BANGLADESH

## Abstract

*Kando* is a Japanese term referring to a spectrum of reactions associated with feeling moved experiences. This study developed an instrument to measure an individual’s degree of *kando* and its associated reactions and explored how Japanese people experience *kando* in their lives. As part of a large-scale survey, we analyzed data from 4,690 Japanese participants aged 20–69. In the survey, participants recalled and described their most significant *kando* events and rated their experiences on a provisional *kando* reaction scale consisting of 43 items. The results indicated that the most significant *kando* events could be grouped into eight clusters: family issues, interpersonal relationships, arts, sports, travel/nature, negative issues, achievements, and religion/disaster. Using exploratory and confirmatory factor analyses, we constructed a 33-item *kando* reaction scale with a correlated 11-factor structure. The degree of *kando* and its relevant reactions differed as functions of event clusters and population characteristics such as sex and age. For example, *kando* on family issues generated multiple, somewhat contrasting, responses such as happiness and hardship. Intense arousal when experiencing *kando* tended to increase depending on the individual’s age. Our *kando* reaction scale can be a useful instrument for describing and exploring psychological mechanisms relevant to *kando*.

## Introduction

When we hold a newborn baby in our arms or see someone winning a sports event after overcoming a slump, we experience unique feelings that move us. In Japanese, this psychological phenomenon is known as “*kando*” [1]. Western countries have explored concepts relevant to *kando* in studies on *kama muta*, being moved, and awe. This study aimed to understand the structure of *kando* among the Japanese. By doing so, we provide a fundamental basis for exploring the universal and cross-cultural underpinnings of the experiences described as *kando* (in Japan), *kama muta*, being moved, and awe (in Western countries).

Psychological research on *kando* has been conducted in Japan since the late 1990s, including a series of studies by Tokaji (e.g., [2, 3]). Tokaji [3] described *kando* as “the state of being emotionally moved.” However, *kando* is not just a psychological phenomenon captured within the conventional framework of emotion research such as the basic emotion theory [4] and the dimensional theory of emotion [5]. Tokaji [3] argued that *kando* occurs in association with both positive (e.g., happiness and joy) and negative (e.g., sadness) emotions. For example, sad experiences associated with *kando* (e.g., bereavement and death) can be accompanied by positive psychological aspects that enable one to overcome poignant experiences. In addition, *kando* can be elicited at various intensities from subtle daily events (e.g., background music on the street) to major life accomplishments (e.g., marriage and childbirth) [6]. The degree of arousal in relation to *kando* ranges from weak to strong and people can experience *kando* with gentle tears or severe goosebumps.

When *kando* is experienced, physical reactions occur alongside the emotional responses. Previous studies [7] have demonstrated that *kando* while listening to music is accompanied by five physical reactions: “goosebumps,” “lump in the throat,” “shiver down the spine,” “tears,” and “arousal.” These psychophysiological reactions have been also reported in the studies of *kama muta*, being moved, and awe [8]. In the following section, we discuss these concepts, summarize the relevant literature, and identify their similarities and differences.

*Kama muta* is a Sanskrit word meaning “moved by love” [9]. Fiske et al. [9] suggested that *kama muta* occurs when communal sharing relationships (hereafter CSRs) with other people or things suddenly intensify. CSRs refer to relationships in which people feel a sense of connection through love, unity, fusion, harmony, patriotism, and identity. For example, when CSRs are rapidly strengthened by witnessing others’ kind behavior, *kama muta* can be experienced. In other words, the trigger for the *kama muta* experience is primarily prosocial or altruistic behavior [10].

Research on the experience of being moved among German nationals identified six event categories that trigger emotional responses: human relationships, significant life events, political and social events, nature-related events, art-related events, and the others (misc.) [11]. Theoretically, such experiences can occur when positive core values fundamentally important for humans are prominent (e.g., brotherhood, solidarity, peace, health, excellence, and beauty) [12]. In addition, Menninghaus et al. [11] showed that events evoking moving experiences are characterized by low-to-moderate levels of arousal, whereas the experience itself is powerful, regardless of the arousal level of the event.

Awe is a psychological state that occurs when one is exposed to a great work of art, intellectual inspiration, the beauty of nature, etc. [13]. It is characterized by its central concepts of “vastness” and “accommodation” [14]. Vastness refers not only to size in the physical sense but also to size in the social sense, including fame, authority, and prestige. In other words, vastness refers to one’s perception of the degree of greatness compared to the presence of the self or internal standards. Accommodation refers to the need to change and adapt one’s cognitive framework when confronting such an amazing entity [15]. Awe, which is a powerful emotion that can change one’s cognitive framework, arises from feelings experienced when encountering an extraordinary presence [16].

The concepts of *kama muta*, being moved, and awe broadly overlap with *kando*. For example, *kando* can be triggered by prosocial or altruistic behavior [17] in the same manner as *kama muta*. We sometimes experience and express *kando* as a strong psychological reaction such as being moved or feeling a sense of awe. However, Yasuda et al. [1] noted that *kando* differs from these concepts. For example, *kando* frequently occurs in contexts that do not involve social or interpersonal relationships such as listening to unfamiliar music or experiencing the majesty of nature. In addition, while the reactions to being moved and awe are powerful, *kando* occurs with a wide range of intensities, from gentle heartwarming *kando* to powerful heart wrenching *kando*. Therefore, despite some overlaps, it is more reasonable to regard *kando* as an umbrella concept that includes *kama muta*, being moved, and awe, as suggested by Yasuda et al. [1].

The present study bases its theory of *kando* on the work of Zickfeld et al. [18], who constructed the KAMMUS II (the *kama muta* multiplex scale) to measure *kama muta*, surveyed 19 countries, and found that *kama muta* is applicable across various cultures and languages. In the KAMMUS II, the “emotion labels” subscale consists of the items “It was heartwarming,” “I was moved,” and “I was touched,” which can be used to represent a general response to *kama muta*. Interestingly, in the KAMMUS II, the item “I was moved” was translated into Japanese using the word “*kando*”; however, the emotion labels score for the Japanese sample was the lowest among the countries in the study. One possible reason is that the concepts of being moved in English and *kando* in Japanese are not concordant. Therefore, it is necessary to conduct a more in-depth assessment of *kando* and comprehend its conceptual framework. This will enable us to determine whether the findings of Zickfeld et al. [18] were attributable to semantic discrepancies between being moved and *kando* or to differences in cognitive processes among participants from Japan and other countries.

As aforementioned, *kando* involves various concepts such as *kama muta*, being moved, and awe. An instrument that measures the experience of *kando* and the relevant concepts is required to confirm whether *kando* is an umbrella term that encompasses these concepts. Therefore, the primary purpose of this study is to construct a *kando* reaction scale (KRS) that measures the broad concept of *kando*, thereby enabling us to understand the characteristics and relevance of its concepts. The literature on *kando* among the Japanese population has focused on particular targets such as films [3], novels [19], and music [7]. However, the KRS should be comprehensively applied to broader situations. We would like to understand how the characteristics of *kando* vary according to the events and targets that trigger them in order to broaden the scope of *kando* that can be measured and consequently comprehend the “general” and “event-specific” components of *kando* in further work.

Our survey had three key aims. First, we sought to examine the types of events that Japanese people memorize as significant *kando* experiences. While we acknowledge that *kando* can be experienced through small, everyday occurrences, we addressed the participants’ “most significant *kando* events” in this study to focus on representative reactions to *kando* experiences. Second, we constructed a KRS to measure the degree of *kando* and the relevant reactions. Finally, we examined the KRS scores across different event categories and demographic factors, including generation and sex. Based on our findings, we developed a suitable instrument to describe and explore the mechanisms of action of *kando*.

## Methods

This study was part of a large-scale research project comprising four quarterly surveys conducted from November 18, 2021 to September 6, 2022. All procedures performed in this study involving human participants were in accordance with the ethical standards of the institutional and/or national research committee and the 1964 Declaration of Helsinki and its later amendments or comparable ethical standards. The survey was approved by the Ethics Review Committee for Research Involving Human Subjects (Humanities and Social Sciences) of Ritsumeikan University (No. Kinugasa-Human-2021–84). Written informed consent was obtained from all participants involved in the study.

### Participants

A total of 13,971 Japanese adults aged 20–69 years participated in one of the four surveys, for which generations (i.e., 20s to 60s) and sexes (i.e., men and women) were assigned equivalently. This study reported the data obtained from the third and fourth surveys, in which a complete set of questionnaire items was incorporated. A survey company conducted the surveys online and obtained data from 6,915 participants in the third (*n* = 3, 445) and fourth surveys (*n* = 3, 470).

#### Exclusion criteria

Some participants in online surveys may be careless because they answer questions without reading the instructions or items carefully (e.g., [20]). Therefore, we included four attention check items (e.g., “Please choose ‘3’ for this item.”) to determine whether participants responded appropriately. Participants with incorrect responses to any of the four items were excluded.

Furthermore, in the surveys, we asked participants to describe their memories of the most significant *kando* experiences (See the procedure for more details). Two assistants individually reviewed all of the descriptions and judged their relevance to *kando*. If an assistant judged the data to be irrelevant, the participants were excluded.

In addition, we asked the participants to choose categories from the 19 items that they felt best represented in their descriptions. We excluded participants who chose seven (i.e., *M* + 3*SD*) or more items because selecting an excessively large number of categories may not accurately represent a single event.

#### Characteristics of the study sample

Data from 2,308 participants (67.0%) in the third survey and 2,382 participants (68.6%) in the fourth survey (*N* = 4, 690) were analyzed. The participants ranged in age from 20 to 69 years (*M* = 45.80, *SD* = 13.73), and there were 2,049 men and 2,641 women. Table 1 provides detailed descriptive statistics of the participants’ ages across sexes and generations.

**Table 1:** Characteristics of the study sample (*N* = 4, 690).

Generation	Sex	*n*	Age	
			*M*	(*SD*)
20s	Men	354	25.64	(2.73)
	Women	449	25.51	(2.76)
30s	Men	375	35.19	(2.82)
	Women	524	34.55	(2.84)
40s	Men	422	45.03	(2.92)
	Women	548	44.72	(2.79)
50s	Men	410	54.70	(2.86)
	Women	563	53.81	(2.81)
60s	Men	488	64.27	(2.88)
	Women	557	63.49	(2.70)
	Total	4690	45.80	(13.73)

### Measures

#### *Kando* reaction scale

The provisional KRS consists of 43 items and was created in Japanese. S1 Table lists all of the items and their English equivalents. The items included general reactions when experiencing *kando* (e.g., “I felt *kando*,” “It stirred my heart,” “It touched my heart”) and adjectives representing emotions (e.g., “It was pleasant,” “I was excited,” “I was happy,” “I was sad”). In addition, considering that the transition from negative/neutral to positive affective states could be relevant to *kando* [1], we included items representing affective transitions (e.g., “While it was happening, I had anxieties and worries, but they were gone by the end”). We also included subjective and physiological reactions reported in the previous studies of *kando* [7] and *kama muta* [18]. Examples of items related to lacrimation are “I cried” and “I was close to tears”; items related to pilomotor reflex are “I had goosebumps” and “I felt a shiver”; and items related to emotional warmth included “There was a warm sensation in my chest” and “It was heartwarming.” Furthermore, regarding *kando*-related concepts [1], we included items related to awe (e.g., “I felt awe”) and spiritual experiences (“I felt a mysterious power”).

#### KAMMUS II

To examine the validity of the KRS, we also included items from the KAMMUS II [18]. The KAMMUS II originally consisted of five sections: sensations (e.g., “moist eyes”), appraisal (e.g., “I observed an incredible bond”), motivation (e.g., “I wanted to hug someone”), valence (e.g., “positive feelings”), and emotion labels (“heartwarming”). However, some of the *kando* events might not be relevant to interpersonal bonds or relationships. In this case, the appraisal and motivation items could not be answered; hence, we excluded them from our survey. The remaining 16 KAMMUS II items overlapped with the provisional KRS because they included the concept of *kama muta*. We translated the KAMMUS II (English version) by ourselves, while Zickfeld et al. [18] provided the Japanese version of KAMMUS II. This is because “being moved” was translated as “*kando*” for the Japanese equivalent, which was inappropriate considering our conceptualization of *kando*. The validity of the translation was confirmed using a professional back-translation service. Owing to this back-translation process, the English expressions in S1 Table differ from the original English version of the KAMMUS II.

### Procedure

First, we asked the participants to recall the most significant *kando* events in their lives. Specifically, we asked them to describe a particular episode and its reactions in over 300 Japanese letters (Detailed analyses of episodes and reactions will be reported in another study). Participants then selected one or more item(s) from the following categories to describe the event: “marriage or romantic relationships,” “pregnancy/childbirth,” “childcare,” “family,” “animals/pets,” “illness/accidents,” “bereavement,” “examination,” “job acquiring,” “sport,” “literary works,” “visual works/arts (including cinemas and television programs),” “music,” “nature/universe,” “disasters,” “travel/cross-cultural experience,” “religion,” “destiny,” and “others (with additional description of details).” These categories were chosen according to the current authors’ discussion based on the categories of Maeura et al. [15], who compared the experiences of awe and *kando*. Subsequently, we asked the participants to complete 13 questionnaires, including the KRS and KAMMUS II. The remaining questionnaires, which mainly examined psychological and subjective well-being, and cultural aspects of human cognition, were used for other studies. The KRS and KAMMUS II were rated on a 7-point Likert scale with the following instruction: “We would like to ask you about what you felt in connection to the most significant *kando* events you have experienced. Please rate the extent to which you felt the following feelings between 1 and 7, with one being ‘Did not feel at all’ and seven being ‘Incredibly strong.”’ The survey was projected to be completed within approximately 15 minutes.

### Data analysis

First, we analyzed the categories selected as participants’ most significant *kando* events. Participants were given the option to choose one or more categories, and their responses were recorded as either 0 or 1. Specifically, a value of 1 was assigned to participants who chose a category, and a value of 0 was assigned to those who did not. We calculated the Jaccard distance between the selected event categories based on coding; that is, the dissimilarity statistic between two binary variables (0 or 1). We conducted a hierarchical cluster analysis (Ward’s method) using Jaccard distances to group similar events, which were then aggregated according to generation and sex.

Next, we conducted an exploratory factor analysis (EFA) and confirmatory factor analysis (CFA) to refine the KRS items. To avoid performing the EFA and CFA on the same sample, we randomly divided the sample (*N* = 4, 690) into two subsamples where the two subsamples had comparable distributions of generations (i.e., 20s–60s) and sexes (men and women). Before performing EFA, we estimated the number of factors using a parallel analysis with squared multiple correlations. This allowed us to estimate the possible maximum number of factors [21] by considering the complex multifaceted nature of *kando*. We then conducted an EFA using the maximal likelihood method and calculated oblimin solutions (oblique rotation). Among the items for which neither factor loading scored > .40, the item with the maximum complexity value was omitted to achieve a simple factor structure. This procedure was repeated until the maximum pattern value for each item reached > .40. We also examined the internal consistency of each factor by computing Cronbach’s *α* coefficient. The EFA was conducted using the psych package [22] in R.

**Fig 1:**
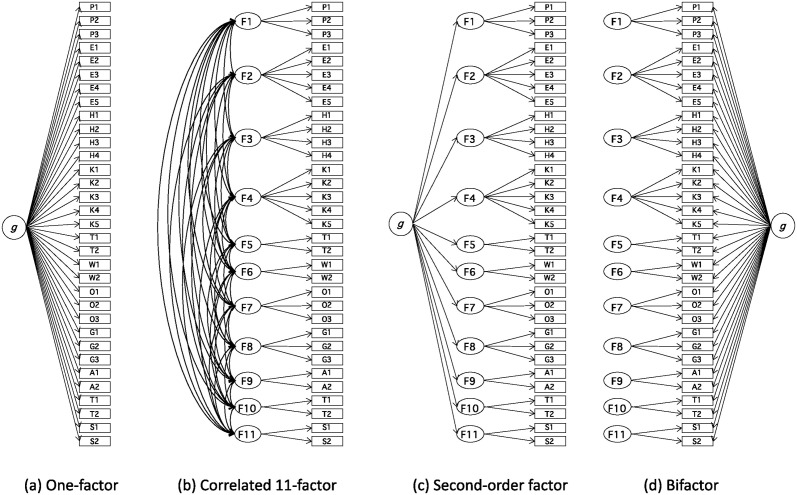
Schematic pictures for four confirmatory factor analysis models. Error variables not depicted.

To examine the KRS factor structure in detail, we conducted a CFA in which we compared four CFA models examined in recent studies (e.g., [23]): (a) one-factor, (b) correlated factors, (c) second-order, and (d) bifactor models (Fig 1). As shown in the schematic (Fig 1), the one-factor model (a) is based on the hypothesis that one general factor (*g*) determines all items. Correlated-factor model (b) indicates that the correlated factors determine the items. The items assigned to each factor were based on the factor patterns determined by the EFA. The second-order model (c) hypothesizes that uncorrelated factors are determined by a second-order general factor (*g*). The bifactor model (d) also hypothesized uncorrelated factors, but the general factor (*g*) loaded onto all the items. Information criteria and fit indices were compared to determine the best-fit model. The cut-off criteria for acceptable fit were based on Hu and Bentler [24]: RMSEA (root mean square error of approximation) < .06, SRMR (standardized root mean squared residual) < .08, CFI (comparative fit index) > .95, and TLI (Tucker Lewis index) > .95. We assessed the validity of the KRS by examining its correlation with the KAMMUS II.

Finally, we analyzed the impact of the cluster of *kando* events on the KRS factor scores to gain insights into the nature of *kando* across various events. As an additional analysis, we examined the effects of participants’ generation and sex on the factor scores to determine whether the degree of *kando* differed depending on the participant demographics. The dataset used in the analyses is available in S1 Dataset.

## Results

### Clustering of most significant *kando* events

**Fig 2:**
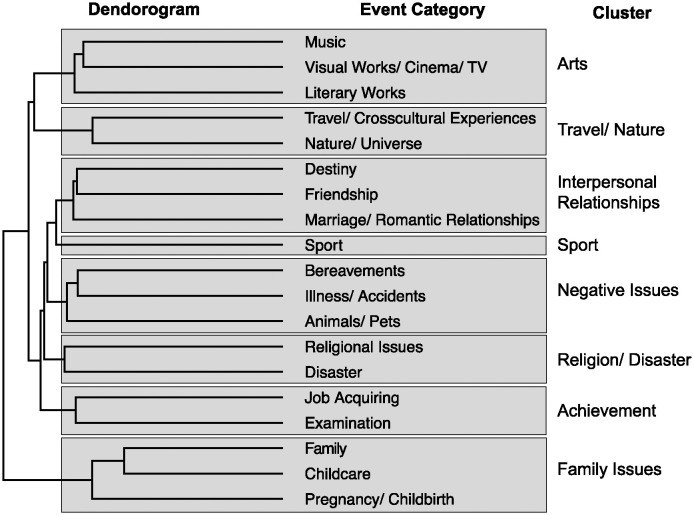
The dendrogram obtained by hierarchical cluster analysis for event categories.

First, we conducted hierarchical cluster analysis (using the Jaccard distance and Ward method) for the categories chosen as the participants’ most significant *kando* events (hereafter called “event categories,” S1 Fig). Based on the dendrogram (Fig 2), we obtained eight clusters, which we named based on the type of event categories: “family issues,” “interpersonal relationships,” “arts,” “sport,” “travel/nature,” “negative issues,” “achievement,” and “religion/disaster.”

We created a cross-table (Table 2) to examine how the distribution of clusters differed according to population characteristics (i.e., generation and sex). In this table, clusters are sorted according to their total frequencies. We conducted a *χ*^2^ test for each cluster to examine the distribution differences among the ten groups (i.e., the combination of five generations and two sexes). Here, we show the categories in each cluster along with the results of *χ*^2^ tests and subsequent residual analyses.

**Table 2:** Cross-tabulation of event clusters by generation (20s to 60s) and sex (M for male, W for female): Results of *χ*^2^ test and residual analysis.

Event Cluster	20s		30s		40s		50s		60s		Total	
	M	W	M	W	M	W	M	W	M	W	M	W
Family Issues	47^−^	149	110^−^	**262^+^**	134^−^	**255^+^**	109^−^	**259^+^**	158^−^	**261^+^**	558	1186
Interpersonal Relationships	**119^+^**	**142^+^**	100	139	95	107^−^	110	125^−^	132	132	556	645
Arts	67	**122^+^**	72	81	55	80	64	78	59^−^	75	317	436
Sport	**79^+^**	21^−^	**78^+^**	27^−^	**83^+^**	47^−^	**98^+^**	54^−^	**103^+^**	42^−^	441	191
Travel/Nature	38	37^−^	41	31^−^	45	78	55	92^+^	72	**100^+^**	251	338
Negative Issues	37	39^−^	29^−^	42^−^	42	65	44	**92^+^**	64	**88^+^**	216	326
Achievement	**49^+^**	34	28	31	40	39	41	26^−^	43	29^−^	201	159
Religion/ Disaster	5	0^−^	5	5	9	5	9	10	**15^+^**	10	43	30
Others	21	24	19	18	32	23	28	21	32	26	132	112

^−^significantly less (*p* < .05); ^+^significantly more (*p* < .05)

The “family issues” cluster (i.e., “family,” “childcare,” and “pregnancy/childbirth”) was chosen the most frequently (27.2% of men and 44.9% of women). The *χ*^2^ test showed a significant association between frequency and group, *χ*^2^(9, *N* = 4, 690) = 227.88, *p* < .001. Furthermore, the residual analysis revealed that there were significantly more women aged 30 years or older in this cluster than the other population groups.

The next cluster (“interpersonal relationships”) was composed of “destiny,” “friendship,” and “marriage/romantic relationships” (27.1% of men and 24.4% of women). In this context, the category of “destiny” refers to accidental, fortuitous, or serendipitous encounters with someone important. Thus, we named this cluster “interpersonal relationships.” The *χ*^2^ test found a significant association between frequency and group, *χ*^2^(9, *N* = 4, 690) = 39.02, *p* < .001. According to the residual analysis, men and women in their 20s belonged to this cluster significantly more than the other population groups.

The “literary works,” “visual works (including cinemas and television programs),” and “music” comprised an “art” cluster. The *χ*^2^ test showed a significant association between frequency and group, *χ*^2^(9, *N* = 4, 690) = 60.49, *p* < .001. The residual analysis indicated that significantly more women in their 20s were in this cluster than the other population groups.

The “sport” category comprised a cluster on its own. The *χ*^2^ test showed a significant association between frequency and group, *χ*^2^(9, *N* = 4, 690) = 213.80, *p* < .001. According to the residual analysis, men of all ages belonged to this cluster significantly more frequently than women.

The categories of “nature/universe” and “travel/cross-cultural experience” comprised a “travel/nature” cluster. The *χ*^2^ test showed a significant association between frequency and group, *χ*^2^(9, *N* = 4690) = 57.95, *p* < .001. The residual analysis showed that women in their 50s and 60s belonged to this cluster significantly more often than the other population groups.

The “negative issues” cluster comprised “bereavements,” “illness/accidents,” and “animal/pets.” We found a significant association between frequency and group, *χ*^2^(9, *N* = 4, 690) = 40.80, *p* < .001. The residual analysis showed that women in their 50s and 60s belonged to this cluster more often than the other population groups.

The “achievement” cluster was comprised of “job acquiring” and “examination.” The *χ*^2^ test yielded a significant association between frequency and group, *χ*^2^(9, *N* = 4, 690) = 39.72, *p* < .001. The residual analysis showed that men in their 20s were significantly more common in this cluster than in other population groups.

Finally, the “religion/disaster” category was identified. Japan frequently experiences natural disasters such as large earthquakes and typhoons. These natural disasters are strong forces that influence the lives of the Japanese people and can be connected to religious thought. The frequency of this cluster showed a significant association with the groups *χ*^2^(9, *N* = 4, 690) = 19.72, *p* = .02. According to the residual analysis, men in their 60s belonged to this cluster significantly more often than the other population groups.

Participants who were not assigned to any cluster were labeled “others” (5.2% [*n* = 243]). Examples of “others” included “volunteer,” “birthday,” “computer games,” “job,” and “hobbies,” hobbies. The association between the frequency of “others” and groups was not significant, *χ*^2^(9, *N* = 4, 690) = 16.52, *p* = .06.

In sum, eight clusters were identified for the participants’ most significant *kando* events. The responses to each cluster differed as a function of the participants’ sex and generation. Sex differences were found in broad generations for the “family issues” and “sport” clusters, which were significantly higher for women (except those in their 20s) and men, respectively. Women of younger ages (20s) responded more to the “arts,” whereas men in this generation did so for “achievements.” Women from the older generations (50s and 60s) responded more to “travel/nature” and “negative issues,” and men in their 60s responded more to the “religion/disaster” cluster.

### Exploratory factor analyses

EFA was conducted to refine the provisional version of the KRS with 43 items. In EFA, we used the first subsample (*n* = 2, 345) chosen from all the samples (*N* = 4, 690). We used a parallel analysis with squared multiple correlations, whereby the number of factors is estimated to be 11. The final pattern matrices are listed in Table 3. The fit indices showed a good fit: *χ*^2^ = 196.81, *p* = .87, RMSR = .01, TLI = .98, RMSEA = .024 (90% CI of the RMSEA [.022,.027]).

**Table 3:** Pattern matrix computed by exploratory factor analysis (EFA) and standardized coefficients computed by confirmatory factor analysis (CFA) based on the correlated 11-factor model.

	Pattern Matrix by Exploratory Factor Analysis													
	F1	F2	F3	F4	F5	F6	F7	F8	F9	F10	F11	H2^a^	Com^b^	CFA^c^
F1 Positive Emotions (*α* = .91 [.90,.91])														
P1	**.90**	.01	-.01	.01	.01	-.01	.01	.03	.00	.04	.02	.86	1.01	.90
P2	**.80**	-.01	-.04	.05	.02	.07	.07	-.02	.04	-.02	.02	.77	1.05	.88
P3	**.67**	.11	.01	-.01	.01	.16	.00	.01	-.06	.01	.02	.72	1.20	.85
F2 Exhilaration (*α* = .84 [.83,.85])														
E1	-.04	**.83**	-.04	.04	.00	.00	-.02	.05	.04	.00	.01	.73	1.02	.79
E2	-.01	**.73**	.04	.03	.02	.01	.07	.03	-.01	.00	.01	.61	1.04	.70
E3	.29	**.49**	-.03	-.08	-.02	.11	-.11	.05	-.08	.07	.09	.60	2.17	.75
E4	.19	**.45**	.07	.02	.05	-.08	.23	.01	-.04	.15	-.10	.50	2.54	.61
E5	.21	**.43**	-.13	.06	-.08	.16	.00	.07	.06	-.07	.07	.56	2.39	.75
F3 Hardship (*α* = .78 [.77,.80])														
H1	.06	-.02	**.77**	.02	-.01	.00	.08	-.05	-.03	.05	-.03	.62	1.06	.75
H2	.00	-.01	**.77**	.01	-.03	-.01	-.09	.08	.08	-.01	.00	.61	1.08	.74
H3	-.06	.03	**.57**	-.14	.04	-.03	-.07	-.01	.06	-.06	.09	.42	1.31	.66
H4	-.18	-.01	**.55**	.04	.06	-.03	.24	-.03	.00	-.04	.01	.53	1.67	.74
F4 *kando* (*α* = .80 [.78,.81])														
K1	-.01	.08	.01	**.68**	.03	.09	-.01	-.01	.01	.03	.02	.62	1.07	.77
K2	-.02	-.06	-.05	**.50**	.06	.16	-.06	.14	.04	.01	.09	.52	1.57	.71
K3	.31	-.04	-.05	**.49**	.08	.01	.02	.09	.01	-.01	-.05	.53	1.93	.67
K4	-.09	.11	.01	**.42**	.05	.17	.05	.00	.01	.07	.09	.40	1.84	.61
K5	.12	.13	.01	**.41**	.04	-.03	.05	.02	.02	.08	.15	.43	1.86	.59
F5 Tears (*α* = .88 [.88,.89])														
Te1	.01	.01	-.01	-.01	**.98**	-.05	-.01	-.02	.01	-.01	-.01	.90	1.01	.87
Te2	.01	-.01	.00	.03	**.79**	.10	-.01	.04	-.02	.02	.00	.74	1.04	.91
F6 Warmth (*α* = .83 [.82,.84])														
W1	.02	.01	-.02	.05	.03	**.78**	.02	-.01	.00	.05	-.02	.73	1.02	.83
W2	.08	.01	.00	.05	.03	**.74**	.00	-.01	-.03	.02	-.01	.69	1.04	.85
F7 Overcome (*α* = .72 [.70,.74])														
O1	.01	.02	-.03	.01	.00	.00	**.84**	.03	.00	-.02	.02	.71	1.01	.80
O2	.08	.01	.09	.01	.00	.02	**.70**	-.02	.01	.09	-.06	.61	1.11	.80
O3	-.06	.01	.02	-.11	.07	.17	**.42**	.06	.09	-.13	.22	.31	2.62	.45
F8 Goosebumps (*α* = .68 [.65,.70])														
G1	.01	-.01	.01	.06	.04	.00	.00	**.74**	-.05	.06	.01	.61	1.04	.72
G2	.04	.13	-.02	-.03	-.05	-.05	.03	**.65**	.07	.03	.03	.57	1.16	.75
G3	-.17	-.05	.15	-.03	.12	.10	.09	**.41**	.13	-.05	.06	.35	2.58	.41
F9 Awe (*α* = .72 [.69,.74])														
A1	.04	-.02	.10	-.02	-.01	-.02	-.01	-.02	**.81**	.01	-.02	.70	1.04	.84
A2	-.03	.04	-.11	.05	.02	.00	.02	.02	**.70**	.05	.01	.51	1.09	.68
F10 Transcendence (*α* = .70 [.67,.72])														
Tr1	.02	-.02	-.01	-.02	.02	.09	-.01	.07	.08	**.68**	-.01	.57	1.09	.68
Tr2	-.01	.10	.01	.12	.03	.04	.05	.12	.06	**.50**	.08	.57	1.48	.77
F11 Surprise (*α* = .58 [.54,.61])														
S1	.13	.02	.01	.09	-.04	-.05	-.03	.12	.02	-.01	**.57**	.47	1.29	.54
S2	-.04	.01	.06	.07	.07	-.02	.06	-.02	.02	.38	**.45**	.52	2.18	.73
Inter-factor Correlations^d^														
F1	—													
F2	.54	—												
F3	-.28	-.15	—											
F4	.36	.28	-.16	—										
F5	.07	.03	.09	.40	—									
F6	.57	.30	-.20	.57	.44	—								
F7	.17	.28	.34	.12	.24	.17	—							
F8	.19	.50	.01	.38	.25	.17	.09	—						
F9	-.11	.08	.36	.11	.09	-.03	.11	.35	—					
F10	.29	.30	.10	.37	.26	.30	.19	.36	.28	—				
F11	.10	.26	.08	.28	.12	.17	.06	.44	.24	.27	—			

Cronbach’s *α* values with 95% confidence intervals are also shown.

^a^H2 indicates commonality.

^b^Com indicates complexity.

^c^CFA indicates the standardized coefficients computed by confirmatory factor analysis.

^d^Inter-factor correlations were computed among factor scores using exploratory factor analysis.

In the following, we describe each item using a combination of the first letter of the factor name and the number (e.g., item 1 of the “positive emotions” factor will be described as “P1”). The list of items is presented in English along with their Japanese originals in S1 Table.

F1 (positive emotions) consisted of P1 (I was joyful), P2 (I was happy), and P3 (I was smiling), indicating positive experiences with the *kando*. F2 (exhilaration) was also positive, but its meaning implied a transition from a negative or neutral mood to a positive one: E1 (I felt exhilarated), E2 (I felt refreshed), E3 (I was cheerful and my feelings were elevated), E4 (I felt a sense of accomplishment), and E5 (It was pleasant). By contrast, F3 (hardship) consisted of the following negative emotions: H1 (I felt anxious), H2 (I felt scared), H3 (It was unpleasant), and H4 (It was emotionally difficult). F4 (*kando*) reflects the general reaction of *kando*: K1 (It stirred my heart), K2 (It touched my heart), K3 (I felt *kando*), K4 (There was some manner of sensation in my chest), and K5 (It was even more moving than initially expected). F5 (tears) indicated physical reactions relevant to tears: Te1 (I cried) and Te2 (I was close to the tears). F6 (warmth) was a construct of heart-warming experiences: W1 (There was a warm sensation in my chest) and W2 (It was heartwarming). F7 (overcome) directly denoted the negative-to-positive changes during an event: O1 (While it was happening, it was difficult and painful, but in the end, it was positive), O2 (While it was happening, I had anxieties and worries, but they were gone by the end), and O3 (Sadness changed to joy). F8 (goosebumps) indicated strong bodily reactions: G1 (I had goosebumps), G2 (I shivered), and G3 (I lost my breath). F9 (awe) reflected the experience of awe: A1 (I felt awe) and A2 (I felt veneration). F10 (transcendence) indicated the experience of a higher power exerting its influence: Tr1 (I felt a mysterious power) and Tr2 (I felt a great power). Finally, F11 (surprise) consisted of two items indicating surprise reactions: S1 (I was surprised) and S2 (It was beyond my understanding).

We also calculated the Cronbach’s *α* coefficients as internal consistency indices (see Table 3) and found that nine of the 11 factors satisfied the cutoff criterion of *α* > .70 as a conventional rule of thumb (e.g., [25]). However, for F8 (goosebumps) and F11 (surprise), the *α* values were lower than.70, perhaps because the *α* coefficients tended to underestimate the internal consistency of the scales consisting of only a few items. In the content analysis, G1 (I had goosebumps), G2 (I shivered), and G3 (I lost my breath) represented strong bodily reactions when experiencing *kando*. The nuances seem to be independent of other factors that measure bodily reactions: F5 (tears) and F6 (warmth). S1 (I was surprised) and S2 (It was beyond my understanding) represented experiences that surpassed participants’ initial expectations. This can be interpreted as internally consistent, expressing a surprise reaction when experiencing a *kando*. The items for the goosebump and surprise factors seem to be internally consistent in their nuances. Therefore, these factors should be included as independent variables in the KRS.

### Confirmatory factor analysis and correlations with the KAMMUS II

CFAs were conducted under the four models shown in Fig 1 using the second subsample of 2,345 participants to examine the validity of the EFA factor structure. We computed the information criteria and fit indices for each model (see Table 4). According to the information criteria (Akaike Information criterion (AIC) and Bayesian Information criterion (BIC)), the correlated 11-factor model indicated the best fit among the four models. For this model (Fig 1b, Table 4b), RMSEA (< .06) and SRMR (< .08) indicated a good fit, whereas the CFI (< .95) and TLI (< .95) indicated an unsatisfactory fit. However, as Perry et al. [26] showed, CFI and TLI are unlikely to meet these criteria when applying a complex model. We believe that the correlated 11-factor model is acceptable for our data and satisfies the combined rule of RMSEA < .06 and SRMR < .09 [24].

**Table 4:** Fit indices and information criteria for four confirmatory factor analysis models.

	*χ* ^2^	*χ*^2^/*df*	RMSEA [90%CI]	CFI	TLI	SRMR	AIC	BIC
(a) One-factor	20,892	1.29	.12 [.11,.12]	.43	.39	.14	290,212	290,592
(b) Correlated 11-factor	**3,844**	**1.27**	**.05 [.05,.05]**	**.90**	**.89**	**.06**	**273,274**	**273971**
(c) Second-order factor	7,534	1.26	.07 [.07,.07]	.80	.79	.11	276,875	277,319
(d) Bifactor	7,122	1.24	.07 [.07,.07]	.81	.79	.11	276,507	277,078

Due to the large sample size of this study, we examined the possible biasness of the CFA model. We conducted CFA for the correlated 11-factor model and computed goodness-of-fit indices (Table 5) for each of the ten population groups (i.e., men and women for the age groups of 20s–60s). As shown in Table 5, the fit indices were mostly equivalent to the CFA model for the overall data (see Table 4b), suggesting the least model biasness.

**Table 5:** Fit indices computed for each of the population groups (generation and sex). M for men and W for women.

		RMSEA [90%CI]	CFI	TLI	SRMR
20s	M	.06 [.06,.07]	.89	.86	.07
	W	.06 [.06,.07]	.89	.87	.07
30s	M	.06 [.06,.07]	.89	.87	.07
	W	.06 [.06,.07]	.89	.86	.07
40s	M	.06 [.05,.06]	.91	.89	.06
	W	.07 [.06,.07]	.87	.84	.08
50s	M	.06 [.05,.06]	.91	.90	.07
	W	.06 [.05,.06]	.91	.90	.06
60s	M	.06 [.06,.07]	.89	.87	.07
	W	.06 [.06,.07]	.89	.87	.07

Next, we examined the validity of the KRS by computing the Pearson’s correlation coefficients between the KRS and KAMMUS II. Hereafter, we report analyses using the merged sample (*N* = 4, 690). The KRS scores were computed as factor scores under the 11-factor CFA model (see S2 Table for KRS inter-factor correlations). For KAMMUS II, we computed subscale scores by calculating the mean values of the member items for each factor. Owing to the large sample size (*N* = 4, 690), even negligible correlation coefficients were significant; therefore, we do not report the coefficients’ significance tests here. In the following, we regard |*r*| > .40 as a substantial correlation under the assumption that these two questionnaires are aimed at measuring the similar concepts of *kando* and *kama muta*. Note that all 16 items of the KAMMUS II overlapped with those of the KRS; therefore, some of the correlation coefficients were very close to 1.00 when the factors shared the same items.

Table 6 shows Pearson’s correlation coefficients between the KRS factor scores and KAMMUS II subscale scores. The *kando* factor (F4) showed positive correlations with all but the *choke-up* (*r* = .36) subscales in KAMMUS II, indicating that the *kando* factor is generally relevant to the reactions measured by KAMMUS II. Similarly, the KAMMUS II *emotion labels* score (i.e., the composition of “being moved,” “being touched,” and “heartwarming”) correlated with most factors in the KRS, except *hardship*, *overcome*, *goosebumps*, and *awe*.

**Table 6:** Pearson’s correlation coefficients between the KRS and the KAMMUS II.

KRS	KAMMUS II						
	Sensations						
	*Tears*	*Chills*	*Warmth*	*Choked up*	*Exhilaration*	*Valence*	*Emotion label*
F1 Positive emotions	.15	.24	**.53**	-.08	**.84**	**.91**	**.54**
F2 Exhilaration	.09	**.55**	**.45**	.12	**.87**	**.64**	**.46**
F3 Hardship	.09	.01	-.17	.33	-.32	-.35	-.24
F4 *Kando*	**.52**	**.46**	**.79**	.36	**.57**	**.50**	**.91**
F5 Tears	**.99**	.23	**.47**	**.40**	.17	.16	**.50**
F6 Warmth	**.47**	.17	**.82**	.12	**.63**	**.65**	**.84**
F7 Overcome	.28	.17	.28	.23	.36	.28	.23
F8 Goosebumps	.29	**.94**	.33	**.56**	**.44**	.20	.39
F9 Awe	.06	.36	.03	**.42**	-.04	-.14	.04
F10 Transcendence	.36	**.56**	**.53**	**.45**	**.50**	.36	**.58**
F11 Surprise	.29	**.59**	**.44**	**.51**	.39	.25	**.50**

Values in bold indicate |*r*| > .40.

In terms of subscale correlations, the KRS scores for positive feelings measured by *positive emotions* (F1), *exhilaration* (F2), and *warmth* (F6) were highly correlated with those in the KAMMUS II (i.e., *warmth*, *exhilaration*, and *valence*), as initially predicted. KRS *exhilaration* (F2) and *warmth* (F6) also correlated with the KAMMUS II *chills* and *tears*, respectively. Regarding subjective bodily reactions, KRS *tears* (F5) is highly correlated with KAMMUS II *tears*, *warmth*, and *choked up*, whereas KRS *goosebumps* (F8) was highly correlated with KAMMUS II *chills*, *choked up*, and *exhilaration*. KRS *awe* (F9) was correlated only with the KAMMUS II *choke-up*. KRS *transcendence* and *surprise* exhibited similar tendencies and were highly correlated with KAMMUS II *chills*, *warmth*, and *choked up*. KRS *transcendence* also showed a high correlation with the KAMMUS II *exhilaration*. In contrast, the KRS *hardship* (F3) and *overcome* (F7) factors were not highly correlated with any of the KAMMUS II factors. This indicates that these factors are not necessarily concordant with positive feelings (i.e., *warmth*, *exhilaration*, *valence*) or other factors in *kama muta*.

We developed the KRS with 33 items under the 11 correlated factors model, which showed an acceptable fit to the current data. Some of the factor scores showed high correlations with the relevant scales in *kama muta* (KAMMUS II), demonstrating the validity of the KRS in measuring the concept of *kando* regarding feeling moved. In contrast, the KRS *hardship*, *overcome*, and *awe* factors were not highly correlated with the KAMMUS II, indicating differences between the KRS and the KAMMUS II. Furthermore, the KRS *transcendence* and *surprise* factors were not included in the KAMMUS II, and these factors showed broad correlations with multiple subscales in the KAMMUS II. This indicates that the KRS extends the KAMMUS II and can be used to measure the degree of *kando* and its relevant reactions, including *kama muta*, awe, and being moved.

### The KRS factor scores across event clusters

**Fig 3:**
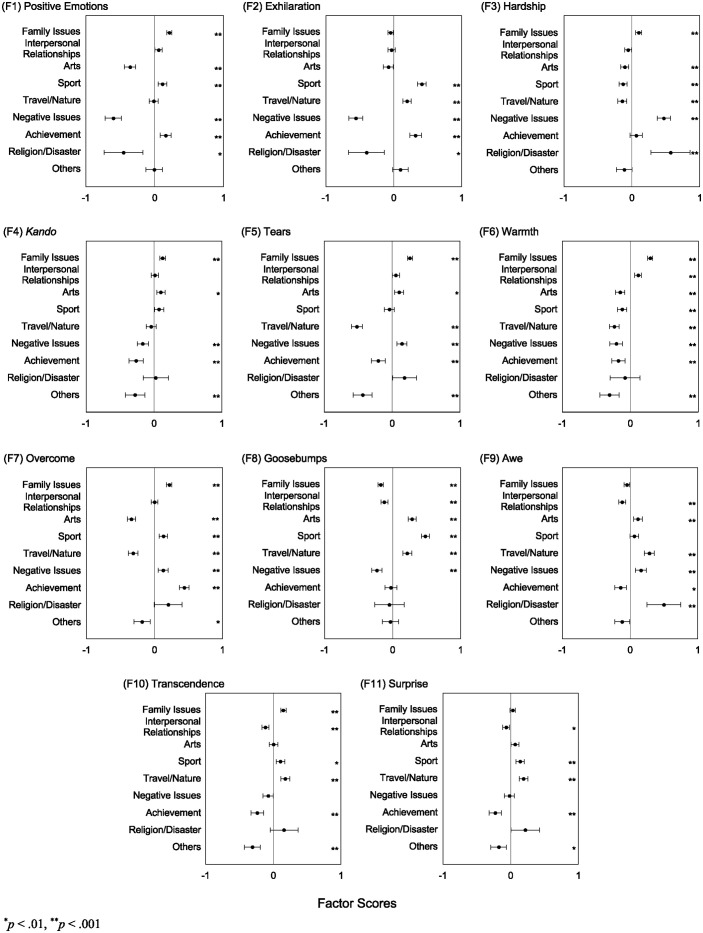
Mean factor scores of each of the KRS factors computed for each event cluster. Error bars indicate 95% confidence intervals of means.

We examined how the KRS factor scores differed as a function of event clusters. We computed the mean values and their 95% confidence intervals to examine the scores across the different event clusters (Fig 3). Note that participants belonged to multiple event clusters; for example, some participants could be assigned to the “family issues” and “arts” clusters when describing their children’s events relevant to music. In other words, the data for different event clusters were not independent; therefore, an analysis of variance (ANOVAs) could not be performed. Alternatively, we conducted one-sample *t*-tests against “0” for each of the event clusters because the factor score is relative to the grand mean (“0”). To adjust for the possibility of a Type I error, we treated *α* = .01 as statistically significant. We primarily reported the KRS factor scores that were significantly higher than the grand mean (“0”) in Table 7.

**Table 7:** Results of one-sample *t*-tests for the KRS factor scores for each event cluster.

KRS	Interpersonal								
	Family Issues			Relationship			Arts		
	(*n* = 1744)			(*n* = 1201)			(*n* = 753)		
	*t*	*p*	*d*	*t*	*p*	*d*	*t*	*p*	*d*
F1 Positive emotions	11.25**	<.001	0.27	2.09	.04	0.06	-8.57**	<.001	-0.37
F2 Exhilaration	-2.33	.02	-0.06	-1.23	.22	-0.04	-2.11	.04	-0.35
F3 Hardship	4.48**	<.001	0.11	-2.18	.03	-0.06	-3.52**	<.001	-0.45
F4 *kando*	6.12**	<.001	0.15	0.45	.66	0.01	3.18*	.002	0.03
F5 Tears	13.84**	<.001	0.33	2.07	.04	0.06	3.06*	.002	0.24
F6 Warmth	15.73**	<.001	0.38	4.53**	<.001	0.13	-4.37**	<.001	-0.08
F7 Overcome	10.85**	<.001	0.26	-0.01	.99	-0.00	-10.63**	<.001	-0.23
F8 Goosebumps	-8.62**	<.001	-0.21	-4.65**	<.001	-0.13	9.44**	<.001	0.05
F9 Awe	-2.27	.02	-0.05	-4.62**	<.001	-0.13	3.51**	<.001	0.46
F10 Transcendence	7.21**	<.001	0.17	-4.45**	<.001	-0.13	0.07	.95	0.18
F11 Surprise	1.46	.14	0.04	-2.77*	.006	-0.08	2.14	0.03	0.23
KRS	Sport			Nature			Negative Issues		
	(*n* = 632)			(*n* = 589)			(*n* = 542)		
	*t*	*p*	*d*	*t*	*p*	*d*	*t*	*p*	*d*
F1 Positive emotions	3.40	.001	0.14	-0.47	.64	-0.02	-10.07**	<.001	-0.43
F2 Exhilaration	12.84**	<.001	0.51	6.18	<.001	0.26	-10.70**	<.001	-0.46
F3 Hardship	-4.34**	<.001	-0.17	-4.36	<.001	-0.18	9.41**	<.001	0.40
F4 *kando*	2.12	.03	0.08	-1.15	.25	-0.05	-3.83**	<.001	-0.17
F5 Tears	-1.10	.27	-0.04	-12.40**	<.001	-0.51	3.74**	<.001	0.16
F6 Warmth	-3.41*	.001	-0.14	-6.36**	<.001	-0.26	-4.40**	<.001	-0.19
F7 Overcome	4.05**	<.001	0.16	-8.51**	<.001	-0.35	3.37*	.001	0.15
F8 Goosebumps	16.26**	<.001	0.65	6.45**	<.001	0.27	-6.12**	<.001	-0.26
F9 Awe	1.92	.06	0.08	7.51**	<.001	0.31	3.87**	<.001	0.17
F10 Transcendence	3.16*	.002	0.13	5.27**	<.001	0.22	-1.98	.05	-0.09
F11 Surprise	4.46**	<.001	0.18	5.81**	<.001	0.24	-0.50	.62	-0.02
KRS	Achievement			Disaster			Others		
	(*n* = 360)			(*n* = 73)			(*n* = 244)		
	*t*	*p*	*d*	*t*	*p*	*d*	*t*	*p*	*d*
F1 Positive emotions	3.85**	<.001	0.20	-3.18*	.002	-0.37	-0.13	.89	-0.01
F2 Exhilaration	7.38**	<.001	0.39	-2.98*	.004	-0.35	1.63	.10	0.10
F3 Hardship	1.38	.17	0.07	3.87**	<.001	0.45	-1.89	.06	-0.12
F4 *kando*	-4.90**	<.001	-0.26	0.26	.80	0.03	-3.81**	<.001	-0.24
F5 Tears	-3.94**	<.001	-0.21	2.01	.05	0.24	-6.06**	<.001	-0.39
F6 Warmth	-3.53**	<.001	-0.19	-0.70	.49	-0.08	-4.23**	<.001	-0.27
F7 Overcome	11.86**	<.001	0.63	1.92	.06	0.23	-3.03*	.003	-0.19
F8 Goosebumps	-0.55	.58	-0.03	-0.42	.67	-0.05	-0.53	.60	-0.03
F9 Awe	-3.30*	.001	-0.17	3.90**	<.001	0.46	-2.10	.04	-0.13
F10 Transcendence	-4.89**	<.001	-0.26	1.52	.13	0.18	-5.21**	<.001	-0.33
F11 Surprise	-4.89**	<.001	-0.26	1.99	.05	0.23	-3.04*	.003	-0.20

*p < .01,

**p < .001

For the “family issues” cluster, higher scores than the grand mean were shown broadly in *positive emotions*, *hardship*, *kando*, *tears*, *warmth*, *overcome*, and *transcendence* factors. In contrast, the “interpersonal relationships” cluster scored significantly high only for the *warmth* factor. For the “arts” cluster, the scores for *kando*, *tears*, *goosebumps*, and *awe* were significantly high. The “sport” cluster also scored significantly high in a variety of factors: *positive emotions*, *exhilaration*, *overcome*, *goosebumps*, *transcendence*, and *surprise*. The “travel/nature” cluster showed similar tendencies in that the cluster scored significantly high for *exhilaration*, *goosebumps*, *transcendence*, and *surprise*; this cluster also scored high for the *awe* factor. In the “negative issues” cluster, the scores were significantly high for *hardship*, *tears*, *overcome*, and *awe*. The “achievement” cluster was similar to the “sport” and “travel/nature” in that the cluster scored significantly high for *positive emotions*, *exhilaration*, and *overcome*. The “religion/disaster” cluster exhibited high scores for *hardship* and *awe*. None of the factors were significantly high for “others.” In summary, we demonstrate that KRS factors can differentiate participants’ experiences among different event clusters, indicating that KRS can describe the characteristics of *kando* for different life events.

### KRS factor scores across populational characteristics

**Fig 4:**
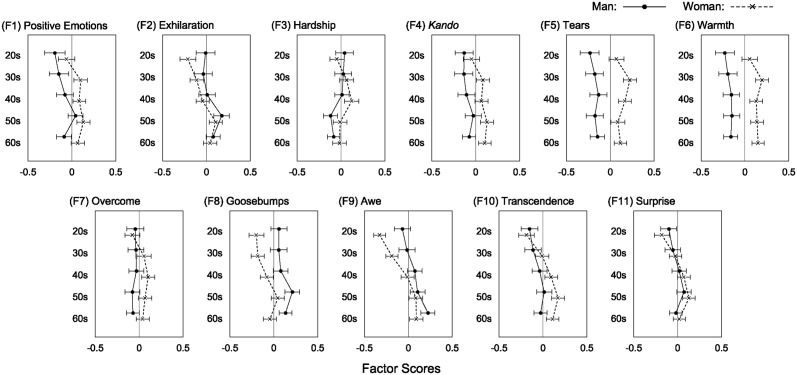
Mean factor scores of the KRS factors across age and sex. Error bars indicate 95% confidence intervals of means.

Finally, we examined how KRS factor scores differed according to the participants’ generation and sex. Fig 4 illustrates the mean values of each KRS factor score by plotting the interactions between generation and sex. We conducted two-way between-subjects ANOVAs with generation and sex as independent variables and each KRS factor score as a dependent variable (Table 8). We also regarded *α* = .01 as the significance level because of the large sample size (*N* = 4, 690).

**Table 8:** Results of analyses of variance for the KRS factor scores dealing the sex and generation as between-subject factors.

KRS	Generation			Sex			Generation × Sex		
	*F*(4, 4, 680)	*p*	$\eta_{p}^{2}$	*F*(1, 4, 680)	*p*	$\eta_{p}^{2}$	*F*(4, 4, 680)	*p*	$\eta_{p}^{2}$
F1 Positive emotions	5.51**	<.001	.005	31.21**	<.001	.007	0.82	.51	<.001
F2 Exhilaration	10.50**	<.001	.009	9.63*	.002	.002	1.06	.38	<.001
F3 Hardship	3.74*	.005	.003	3.16	.08	<.001	1.62	.17	.001
F4 *kando*	2.75	.03	.002	34.79**	<.001	.007	0.59	.67	<.001
F5 Tears	1.82	.12	.002	123.56**	<.001	.026	0.84	.50	<.001
F6 Warmth	1.31	.27	.001	131.52**	<.001	.027	0.54	.71	<.001
F7 Overcome	1.32	.26	.001	11.42**	<.001	.002	1.31	.26	.001
F8 Goosebumps	7.78**	<.001	.007	57.69**	<.001	.012	0.56	.69	<.001
F9 Awe	24.37**	<.001	.020	20.05**	<.001	.006	1.80	.05	.002
F10 Transcendence	11.64**	<.001	.010	14.64**	<.001	.003	1.55	.18	.001
F11 Surprise	9.75**	<.001	.008	0.67	.41	<.001	0.89	.47	<.001

*p < .01,

**p < .001

The ANOVAs showed that the two-way interactions were not significant (*ps* > .01) for either factor. The main effects of generation were significant for the factors of *positive emotions* (F1), *exhilaration* (F2), *hardship* (F3), *goosebumps* (F8), *awe* (F9), *transcendence* (F10), and *surprise* (F11). However, the effect sizes were small or negligible ($\eta_{p}^{2}\leq .01$), except for the small to medium effect of awe ($\eta_{p}^{2}=.02$). For the factors excluding *hardship* (F3), values increased in individuals in their 20s–50s and plateaued or decreased in individuals in their 60s (Fig 4). According to multiple comparisons using Bonferroni’s correction, participants in their 20s showed significantly lower scores for *positive emotions* (than those in their 40s and 50s), *exhilaration*, and *goosebumps* (than those in their 50s and 60s), as well as *awe*, *transcendence*, and *surprise* (compared to those in their 40s, 50s, and 60s). Participants in their 30s also exhibited lower scores for *exhilaration* and *goosebumps* (compared to those in their 50s and 60s), *awe* (compared to those in their 40s, 50s, and 60s), *transcendence*, and *surprise* (compared with those in their 50s). Participants in their 40s scored significantly lower for *exhilaration* and *goosebumps* than those in their 50s but scored significantly higher for *hardship* than those in their 50s and 60s. The scores were not significantly different between participants in their 20s and 30s. The differences between those in their 50s and 60s were not significant, except that the *surprise* score was significantly higher for those in their 50s than for those in their 60s. These results show that the generational effects in the *kando*-related reactions of *positive emotions*, *exhilaration*, *goosebumps*, *awe*, *transcendence*, and *surprise* generally increase with age, peaking around the 50s. The *hardship* experience was highest for those in their 40s and decreased for those in their 50s and 60s.

The main effects of sex were significant for *positive emotions* (F1), *exhilaration* (F2), *kando* (F4), *tears* (F5), *warmth* (F6), *overcome* (F7), *goosebumps* (F8), *awe* (F9), and *transcendence* (F10). However, the effect sizes were small or negligible ($\eta_{p}^{2}\leq .01$) for most factors, except for the small-to-medium effects of *tears* ($\eta_{p}^{2}=.026$), *warmth* ($\eta_{p}^{2}=.027$), and *goosebumps* ($\eta_{p}^{2}=.012$). As shown in Fig 4, men scored significantly higher on *exhilaration*, *goosebumps*, and *awe*, whereas women scored significantly higher on *positive emotions*, *tears*, *warmth*, *overcome*, and *transcendence*.

## Discussion

This study explored the types of events that Japanese people memorized as their most significant *kando* experience and examined how they reacted to them according to the newly developed KRS. We developed the KRS with 11 correlated factors by applying EFA and CFA, which included emotional states (i.e., *positive emotions*, *exhilaration*, *hardship*, *overcome*, *surprise*); subjective physical reactions (i.e., *goosebumps*, *warmth*, *tears*); and the general experience or perception of the event (i.e., *kando*, *transcendence*, *awe*). We also showed that the KRS scores differed as a function of the event’s characteristics: family issues, interpersonal relationships, arts, sports, travel/nature, negative issues, achievements, religion/disaster, and others. The generational effects on *kando* were not overly significant, but *kando*-related reactions with strong arousal (i.e., *positive emotions*, *exhilaration*, *goosebumps*, *awe*, *transcendence*, and *surprise*) were likely to be gradually enhanced for participants in their 20s and 50s. The effects of sex were found to be relatively stronger for subjective physical reactions (*warmth*, *tears*, and *goosebumps*) than for psychological reactions.

Notably, we examined the various events experienced by more than 4,000 people. The existing literature on *kando* (e.g., [2, 3, 7, 19]) has focused only on a specific target (e.g., music, novels, stories); therefore, researchers have not examined the reactions among different domains. The current study enabled us to compare reactions among different domains through participants’ recall of events. For example, we found that “interpersonal relationships” and “family issues,” both in the domain of human-to-human relationships, differed in the evaluations of our KRS. This indicates that the KRS is suitable for describing the characteristics of *kando*, at least when dealing with experiences stored in autobiographical memories.

The event categories were grouped into eight clusters. Menninghaus, et al. [11] qualitatively categorized experiences of being moved into six groups among German nationals: “relationship,” “critical life,” “political event,” “nature-related,” “art-related,” and “others (misc.).” Our eight categories overlap those of Menninghaus et al. [11], suggesting that events triggering *kando* and being moved are analogous between Japan and Germany. However, differences between the two studies were also observed in the distribution of categories. For example, the “art-related” category was chosen as the fifth most prominent of six categories in Germany [11]. In contrast, it was the third most prominent factor among the eight clusters in the present study. Although this may be due to differences in concepts between *kando* and being moved, the degree of *kando* can vary cross-culturally, even if the same events can generate *kando*.

We demonstrated that the KRS effectively differentiated the participants’ reactions among event categories. As in KAMMUS II, the KRS contains emotional and physiological reactions, and several new factors in the KRS were identified. First, the KRS contains *hardship* as a negative emotion, extracted independently of positive emotions. As discussed in the literature on affective well-being [27], negative emotions are not the opposite of positive ones. Tokaji [3] suggested that *kando* is associated with complex and mixed emotions. Similar to our data, family issues scored high in both the *positive emotions* and *hardship* factors, providing evidence that positive and negative emotions (i.e., *hardship*) should be measured independently to describe the entire *kando* experience.

In addition, the *overcome*, *surprise*, and *transcendence* factors were identified for the first time in this study and have not been included in existing scales, such as the KAMMUS II. For events involving relatively long processes, such as family issues, sports, negative issues, and achievements, people responded more strongly to the *overcome* factor, which represents an affective transition from negative to positive experiences, evidencing that *kando* is positive overall despite occasional negative events [1]. Although *kama muta* includes these affective transitions, as represented by the phrase “tears of joy” [8], we explicitly included this as a *overcome* factor in the KRS.

The *transcendence* factor was uniquely extracted in this study. We identified the “religion/disaster” category as one type of event triggering *kando*, suggesting that the meaning of *kando* also encompasses the unknown or religious issues (i.e., “I felt a mysterious power,” “I felt a great power”). In the literature on strong music experiences, people in Western countries feel connected to God through music [28]. To the best of our knowledge, such a *transcendence* factor has not been examined in the research on being moved or *kama muta*. Using a cross-cultural study, it may be beneficial to explore whether this factor is unique to *kando* in Japan (or Asian/Buddhist countries) or whether the mental mechanism also exists in people in Western countries.

Despite the smaller effect sizes, we identified differences in the KRS based on age and sex. Generational effects were particularly prominent in the KRS factors associated with strong arousal (i.e., *positive emotions*, *exhilaration*, *goosebumps*, *awe*, *transcendence*, and *surprise*). Research on emotions and memory has shown that people tend to remember high-arousal experiences more frequently than low-arousal ones [29]. Moreover, memories are likely to be reinterpreted upon recall, and people tend to transform negative experiences into positive ones (i.e., autobiographical reasoning [30]). Therefore, participants in their 20s and 50s may be more likely to interpret events positively through repeated recall of memories.

This study also highlighted that sex differences were greater for the subjective physical reactions of *tears*, *warmth*, and *goosebumps* than for psychological reactions. Considering women’s higher biological vulnerability to adverse and stressful events [31], it is noteworthy that they are more responsive to *kando* events with *tears* and *warmth*. In contrast, *goosebumps* was rated higher by men. The meaning of goosebumps (*torihada* in Japanese) originates from piloerection owing to low temperatures and has been used analogically when describing fear, disgust, nervousness, and anxiety [32] and acquired positive connotations in the lexicon after 1990s. The term is frequently used in sports research [32]. Considering that men are more likely to choose the sports event cluster and that the term “goosebumps” is commonly used in sports, higher ratings of *goosebumps* by men might be influenced by sociocultural factors.

Considering the scope of this study, we constructed a CFA model using covariance-based structural equation modeling (CB-SEM). However, further exploration is possible by addressing the causal relationships among the factors using partial least squares structural equation modeling (PLS-SEM) [33]. By doing so, we can explore the mental structure of how the overall evaluation of *kando* is determined by the psychological and physical components. These recent statistical developments will enable us to understand the direct and indirect indicators explaining the degree of *kando*. This may also allow us to refine the KRS into its validated, shorter version.

Finally, this study has several limitations. First, our data were based on people’s memories; therefore, it remains unclear whether the KRS can measure direct reactions to stimuli, especially in a laboratory setting. In addition, because we asked participants to recall the “most significant *kando* event,” we cannot know whether the KRS can be used for recently experienced events. Furthermore, this study was conducted among a Japanese population; therefore, the applicability of the KRS to other populations remains unknown. We suggest cultural roles in the experience of *kando* so that multi-linguistic studies (e.g., [34]) can be conducted in the future using validated translations of the KRS. This would allow us to understand the role of culture in the experience of *kando*.

## Supporting information

S1 Fig Frequency of categories selected as the most significant *kando* events. (EPS)

S1 Table The *kando* reaction scale in Japanese alongside its English equivalents. (PDF)

S2 Table Inter-factor correlations of the *kando* reaction scale computed using confirmatory factor analysis. (PDF)

S1 Dataset Raw data used in all analyses. (XLSX)
