# Supplementary material for: Uncovering the essence of moving experiences in Japanese culture: Development and validation of a kando reaction scale
Source: PLoS One. 2024 Dec 5;19(12):e0311905. doi: 10.1371/journal.pone.0311905 (PMC11620437; doi:10.1371/journal.pone.0311905)
Supplement: S1 Table — (PDF) [file pone.0311905.s002.pdf]

S1 Table. The *kando* reaction scale in Japanese alongside its English equivalents.

Instruction in Japanese: あなたが体験した「人生で最も感動した出来事」に関連してあなた自身が感じたことをお尋ねします。そのときのあなた自身が下記の項目をどの程度強く感じたかを思い出して，1が「全く感じなかった」，7が「非常に強く感じた」となるように選んでください。

Instruction in English: “We would like to ask you about what you felt in connection to “the most significant *kando* events” you have experienced. Please rate the extent to which you felt the following feelings between 1 and 7, with one being ‘Did not feel at all’ and seven being ‘Incredibly strong.

	Japanese Originals	English (back-translated)
P1	喜んだ	I was joyful
P2	嬉しかった	I was happy
P3	笑顔になった	I was smiling
E1	爽快感があった	I felt exhilarated
E2	スッキリした	I felt refreshed
E3	ウキウキして気持ちが軽くなった	I was cheerful and my feelings were elevated
E4	達成感を覚えた	I felt a sense of accomplishment
E5	快かった	It was pleasant
H1	不安を感じた	I felt anxious
H2	恐怖を感じた	I felt scared
H3	不快だった	It was unpleasant
H4	つらかった	It was emotionally difficult
K1	心が動かされた	It stirred my heart
K2	心を打たれた	It touched my heart
K3	感動した	I felt *kando*
K4	胸の中に何らかの感覚があった	There was some manner of sensation in my chest
K5	当初の予想を上回る心の動きがあった	It was even more moving than initially expected
T1	涙が出た	I cried
T2	目が潤んだ	I was close to the tears
W1	胸の中にあたたかい感覚があった	There was a warm sensation in my chest
W2	心があたたまった	It was heartwarming
O1	途中つらいことや苦しいことがあったが，最後にはポジティブなことだった	While it was happening, it was difficult and painful, but in the end, it was positive
O2	途中，不安や心配事があったが，最後には解消された	While it was happening, I had anxieties and worries, but they were gone by the end
O3	悲しみが喜びに変化した	Sadness changed to joy
G1	鳥肌が立った	I had goosebumps
G2	ゾクゾクした	I shivered
G3	息がつまった	I lost my breath
A1	畏怖を感じた	I felt awe
A2	畏敬を感じた	I felt veneration
T1	神秘的な力を感じた	I felt a mysterious power
T2	強大な力を感じた	I felt a great power
S1	驚いた	I was surprised
S2	自分の理解を超えていた	It was beyond my understanding
The following items were excluded from the *kando* reaction scale.		
	悲しかった	I was sad
	興奮した	I was excited
	鎮静した	I felt calm
	覚醒した	I felt awakened
	共感した	I felt empathy
	自分の考え方に変化があった	There was a change in my thinking
	胸が締め付けられた	My chest tightened
	言葉を失った	I was speechless
	元気が出た	I felt uplifted
	時間があっという間に過ぎた	Time passed in the blink of an eye

* Note that the survey in this study was conducted online and the items listed here were randomly presented to participants.
