# Supplementary material for: Uncovering the essence of moving experiences in Japanese culture: Development and validation of a kando reaction scale
Source: PLoS One. 2024 Dec 5;19(12):e0311905. doi: 10.1371/journal.pone.0311905 (PMC11620437; doi:10.1371/journal.pone.0311905)
Supplement: S2 Table — (PDF) [file pone.0311905.s003.pdf]

S2 Table. Inter-factor correlations of the *kando* reaction scale computed using confirmatory factor analysis.

	F1	F2	F3	F4	F5	F6	F7	F8	F9	F10	F11
F1 Positive emotions	1.00										
F2 Exhilaration	.78	1.00									
F3 Hardship	-.42	-.29	1.00								
F4 *Kando*	.59	.58	-.22	1.00							
F5 Tears	.18	.12	.08	.58	1.00						
F6 Warmth	.74	.53	-.31	.80	.52	1.00					
F7 Overcome	.32	.38	.40	.29	.30	.31	1.00				
F8 Goosebumps	.27	.64	.09	.58	.32	.23	.26	1.00			
F9 Awe	-.16	.08	.53	.13	.07	-.11	.20	.49	1.00		
F10 Transcendence	.43	.59	.12	.73	.41	.51	.39	.73	.47	1.00	
F11 Surprise	.31	.50	.20	.67	.33	.37	.33	.75	.48	.88	1.00
